# Editorial: Is the Language Faculty Nonlinguistic?

**DOI:** 10.3389/fpsyg.2016.00861

**Published:** 2016-06-10

**Authors:** Umberto Ansaldo, N. J. Enfield

**Affiliations:** ^1^Department of Linguistics, University of Hong KongHong Kong, China; ^2^Department of Linguistics, The University of SydneySydney, NSW, Australia

**Keywords:** Universal Grammar, development, innateness, evolution, phonology, syntax, semantics

A line of research in cognitive science over several decades has been dedicated to mapping a hypothetically innate, language-specific cognitive system, a faculty that allows human infants to acquire languages natively without formal instruction and within short periods of time. In recent years, this search has attracted significant controversy in cognitive science generally, and in the language sciences specifically. Some maintain that the search has had meaningful results, though there are different views as to what the findings are: ranging from the view that there is a rich and rather specific set of principles, to the idea that the contents of the language faculty are—while specifiable—in fact extremely minimal. Other researchers rigorously oppose the continuation of this search, arguing that decades of effort have turned up nothing. The fact remains that the proposal of a language-specific faculty was made for a good reason, namely as an attempt to solve the vexing puzzle of language in our species. Much work has been developing to address this, and specifically, to look for ways to characterize the language faculty as an emergent phenomenon; i.e., not as a dedicated, language-specific system, but as the emergent outcome of a set of uniquely human but not specifically linguistic factors, in combination. A number of theoretical and empirical approaches are being developed in order to account for the great puzzles of language—language processing, language usage, language acquisition, the nature of grammar, and language change and diversification. The goal of this Research Topic is to ask whether a paradigm shift has indeed occurred that allows us to conceptualize language not as an innate, dedicated faculty, but as the result of general cognitive abilities adapted for linguistic use.

In the first of three review articles, Dąbrowska reviews the fundamental arguments in support of the Universal Grammar hypothesis. The focus is on the three most powerful arguments, namely universality, convergence, and poverty of stimulus. The author maintains that all three can be proven wrong: languages have been shown to display deep differences of structure; significant variation has been documented in speakers' knowledge of grammar; and grammatical constructions have been proven to be learnable through input. The second review by Christiansen and Chater takes issue with the latest, most minimal proposal for a language faculty (LF): recursion. Through a review and discussion of genetic, non-human primate and neuro-scientific research the authors argue that an innate LF is evolutionarily unlikely. The ability to process recursive structure emerges gradually through adaptation of domain-general sequence learning abilities. The relationship between domain-specificity and linguistic adaptation is the focus of the third review, by Culbertson and Kirby. The authors propose that our linguistic knowledge is best seen as a unique interaction of domain-general capacities with language. This can be illustrated by what they see as a powerful general bias towards simplicity of representation, which manifests itself cross-linguistically through universal tendencies such as compositionality, regularity, harmony, and isomorphism.

In their more theoretical article, Mattos and Hinzen shift the focus of the debate to the acquisition of declarative gestures in pre-verbal children. Even before the onset of one-word expressions, children show the ability to link lexical concepts to gestures. This, the authors argue, can only be explained by a system that is both symbolic and referential, and must be taken as a challenge to the alleged non-linguistic roots of natural pedagogy. In the second article of more theoretical nature, Adger and Svenonius defend the view that “aspects of our best theories of syntactic phenomena are simply special cases of more general principles. But those more general principles are not established at the moment […] generative syntax provides a potential way to reach those more general principles.” A methodological point made here is that in evaluating domain specificity we need to ensure that we evaluate principles of actual explanatory power. A theoretical point maintains that principles might exist that are language-specialized, i.e., linguistic versions of more general cognitive principles. The third of these more theoretically oriented contributions, by Goldberg, concerns exactly what kind of evidence should be used in support of UG. Goldberg looks at the “subtle and intricate” implicit knowledge of language that speakers seem to possess. Even these cases, the author argues, do not warrant the positing of unlearned syntactic structures, as they can be explained by the functions of the constructions involved. Crucially these are learned, conventionalized, and only require domain-general constraints on perception, attention and memory.

Two original research papers offer strong views against innateness. Archangeli and Pulleyblank present a take on phonology based on the Emergent Grammar Hypothesis. In this view humans are understood to make sense of linguistic data primarily through three non-linguistic abilities: categorial thinking, sensitivity to frequency, and symbolic generalization. In three case studies ranging from English to Bantu and Esimbi, the authors show how diverse language data can be explained by such operational abilities. They propose an emergent basis for not only phonology but possibly morphological structures too. In a second original research paper, Evans approaches human language as a communicative system that must have two fundamental design features: a conceptual and a linguistic system, each of which contributes to meaning construction. Evans argues that both systems operate in a symbiotic relation and are semantic in nature, but the former is evolutionarily older and is the one to which the latter is adapted.

Finally, in a perspective piece, Everett takes issue in particular with the notion of a “phonological mind,” or phonological nativism. The proposal, according to the author, suffers from at least two shortcomings. A theoretical problem is that properties invoked in phonological nativism are not successfully explained in evolutionary terms. A methodological problem confuses design features of any given system with innate, rather than acquired, constraints.

We are pleased to present a set of articles that approach our research question—Is the language faculty nonlinguistic?—from a range of angles, and with consideration of multiple stances on the question. Perhaps most importantly, this applies to the very idea of what a language faculty is. The concept can be understood in two distinct ways:

1. That which humans have which is biologically necessary to learn language.
2. That which humans have which is biologically necessary to learn language and which is not a general purpose learning mechanism.

We might call (1) an axiom. Nobody *hypothesizes* that humans have a capacity for language. Rather, that capacity is the thing to be explained and understood. By contrast, (2) is a hypothesis, i.e., that the relevant mechanisms are not general but are specifically dedicated to language. These two concepts of a language faculty must not be confused. Progress with this central problem in the psychology of language will not only require a constructive approach to dialogue between those of differing views, it will require conceptual clarity at every step.

## Author contributions

All authors listed, have made substantial, direct, and intellectual contribution to the work, and approved it for publication.

### Conflict of interest statement

The authors declare that the research was conducted in the absence of any commercial or financial relationships that could be construed as a potential conflict of interest.

